# 5G/6G mobile telephone technology, methodical progress, pertinent health protection, and artificial intelligence

**DOI:** 10.3389/fpubh.2026.1847890

**Published:** 2026-09-10

**Authors:** Sunil Jain, Prem Kamal Jain

**Affiliations:** 1 Manipal Tata Medical College, Jamshedpur, India; 2 Department of Computer Science & Applied Bioinformatics, Indraprastha Institute of Information Technology, New Delhi, India

**Keywords:** automation, communication, data analysis, habits, radiation nonionizing, radiation protection, smartphone, wearable electronic devices

## Abstract

Global mobile data traffic is growing rapidly. Artificial intelligence (AI) plays an important role in this growth, and safety should be a priority in all aspects of this development. Adverse effects on human health with widespread use with upcoming 5G/6G mobile networks need to be addressed. AI with its growing capabilities needs to be explored for safety strategies. We elaborate the AI-based safety measures as ‘Conceptual Framework’ comprising “3RRs”: risk-reducing devices, risk-reducing engineering and environment; and risk-reducing personalization/habits. For devices using frequencies <6 GHz, the electromagnetic field (EMF) exposure measure is in terms of “specific energy absorption rate” for devices using frequencies <6 GHz, the EMF is measured as “absorbed power density.” AI is utilized to lower exposure, with sophisticated algorithms analyzing real-time monitoring data. Wireless gadgets used in various fields like mobile communication, health monitoring, exercise monitoring, warfare tele-communications, etc. AI is useful for automating information delivery among multiple smart applications simultaneously and safely, with automatic sequential decision-making in dynamic environments. Engineering environmental safety with reduced radiation is required. The implementation of 6G and future networks, which aim to create a fully connected world, requires dense deployment. This advancement is possible with AI, enabling dynamic deployment, continual adaptation, autonomous decision-making, and robust control systems. Satellite direct-to-device (D2D) communications are only valuable in truly remote areas and in disasters. Personal habits, simple, healthy, safe, enumerated as an ‘ABC’ plan. AI’s capability for real-time data analysis and decision-making can guide users for safe use. Guidance for growth with safety strategies suggested should be useful for progress in engineering, policies of governments’, and people’s healthy habits proactively.

## 1 Introduction

Progressive sophistication and practical safety measures are important. The aphorism “Radiation uses and ubiquitousness: The good, bad, and limits” in a published review cautioned for rational carefulness (1). Furthermore, artificial intelligence (AI) should be utilized for precise and practical safety of humans and the environment, including flora and fauna. Electromagnetic radiation has been categorized as “possibly carcinogenic to humans” by the World Health Organization (2, 3). A recent systematic review commissioned by the WHO as part of the radio frequency electromagnetic field (RF-EMF) Environmental Health Criteria update has highlighted that (i) exposure to RF from mobile phone use likely does not increase the risk of brain cancer (low certainty evidence), (ii) RF from broadcasting antennas or base stations likely does not increase childhood leukaemia risk (moderate certainty evidence), and it may not increase the risk of pediatric brain tumors (low certainty evidence). This review did not assess the association between 5G mobile phone use and neoplasia risk because, given the short time since the introduction of 5G technology, there were no epidemiological studies directly investigating 5G mobile networks (4).

IARC’s Advisory Group to Recommend Priorities in Carcinogenic Hazards to Humans during 2025–2029 identifies RF EMF, including wireless mobile radiation, as high priority (5).

The European Commission’s ‘Scientific Committee on Health, Environmental and Emerging Risks (SCHEER)’ has rightly pointed out the need for more research in the higher frequency bands of the RF spectrum (i.e., millimeter waves) and their adverse, favorable, or lack of health effects (6).

In our present state of evidence and potential risks, prioritizing safety with professional sophistication is prudent. The International Commission on Non-Ionizing Radiation Protection (ICNIRP) has guidelines on Limiting Exposure to Time-Varying Electric, Magnetic and Electromagnetic Fields (100 kHz to 300 GHz) (7).

Artificial intelligence (AI) advancements enable data analysis, desired insights, intelligent decisions, automation, and predictive capabilities, all precisely, speedily, and perfectly. A new era of artificially intelligent self-optimizing networks is promising and should promote safety for health (8).

International Telecommunication Union (ITU) ITU-R reports global mobile data traffic of 158 exabytes/month in 2022. It is projected to reach 2,194 exabytes/month by 2028 and 5,016 exabytes/month by 2030 (Exabyte = 1,024 bytes) (8). AI will play an important role in this delivery, and, throughout, safety should be a progressively higher priority.

Future planning for wireless telecommunications focuses on frequencies above 6 GHz (Giga Hertz) and into the ‘millimeter wave’ range (30–300 GHz/10–1 mm wavelength) (9). These frequencies are very promising due to their availability of large amounts of raw bandwidth and the potential for multigigabit-per-second (Gb/s) data rates. They offer the advantage of fulfilling (i) the increasing demand for higher data rates, (ii) improved quality of service, and (iii) lower latency to users. Frequencies >6 GHz in various applications, such as radar, airport security screening, and medical therapy, have been used for many years. However, with the widespread adoption of upcoming 5G/6G mobile networks, there are concerns about potential harm to human health. Hence, it is necessary to develop safety strategies.

A scientifically safe aspect of high-frequency usage is that body tissue penetration decreases with increasing frequency. The depth of penetration above 6 Gigahertz (GHz) is relatively short, and the predominant effect is surface heating (10). The non-ionizing nature is also a safe aspect. 6G telecommunications will use a frequency range between 95 GHz and 3 THz (Tera Hertz). These are also non-ionizing, despite being three to a thousand times higher than 5G’s frequency (11).

## 2 Conceptual framework

**Figure 1:**
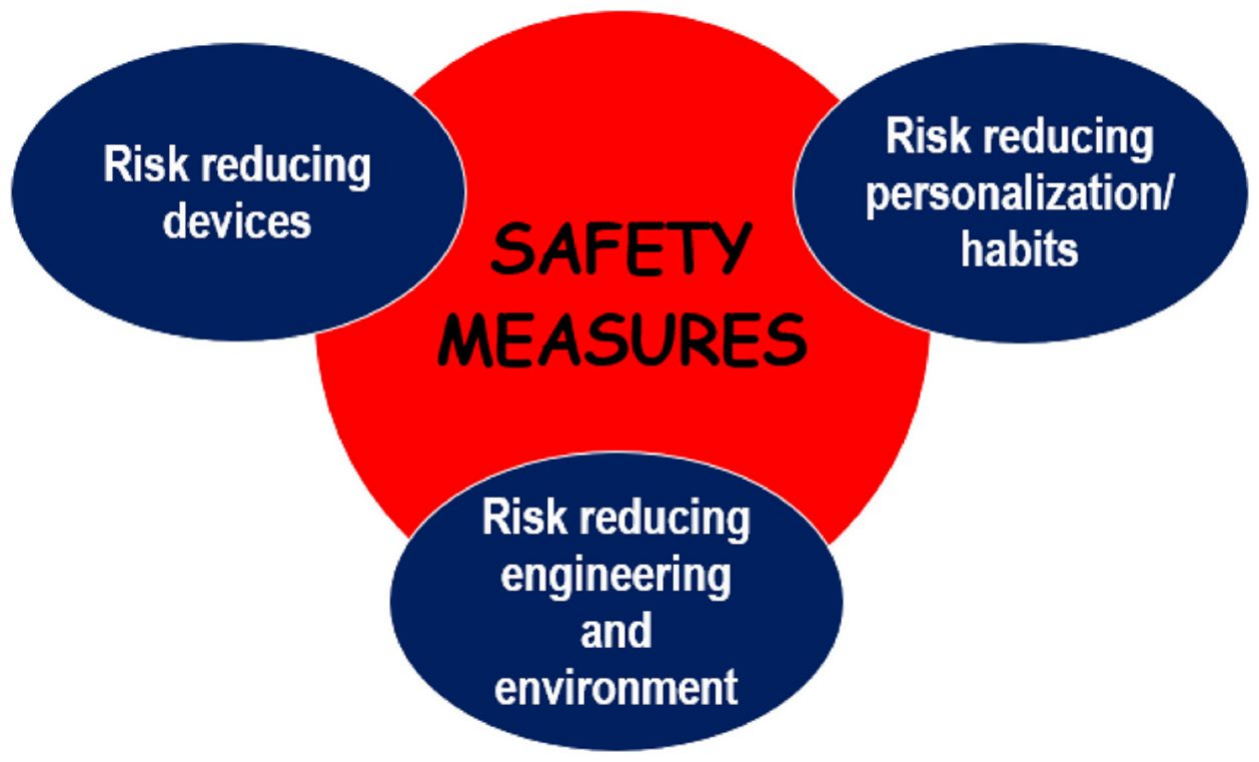
Comprehensive key components for scientific systematic strategies as favourable conceptual framework.

Conceptual frameworks represent ways of thinking about a problem and ways of representing how complex things work (12, 13). We elaborate the AI-based safety measures scientifically and methodically for comprehensive fruitfulness as a ‘Conceptual Framework’ comprising three major components (“3RRs”) (Figure 1):

1. Risk-reducing devices.
2. Risk-reducing engineering and environment.
3. Risk-reducing personalization/habits.

The construct of this is simple. First is the closest source of RF EMF exposure, i.e., the mobile telephone devices. Second is how RF EMF reaches the users, i.e., the environmental engineering. Third is, once technical aspects are taken care of, logically personal use. Further, it is similar but not the same as “3Rs” (replacement, reduction, refinement), which is strongly established in the research ethics literature. Replacement with safer devices, reduction with environmental engineering, and refinements in use.

All AI applications should be compliant with data protection regulations, such as the European Union’s General Data Protection Regulation (GDPR) and the Digital Personal Data Protection Act (DPDPA), 2023 of India.

### 2.1 Risk-reducing devices

The need for safer telephone devices is justified, as mobile phones, rather than transmitting installations, impose the greatest burden of exposure (10). Radiation from user equipment (UE) is more localized and more damaging.

High-level protection for humans from exposure to EMFs from 100 kHz to 300 GHz is prudent. The International Commission on Non-Ionizing Radiation Protection (ICNIRP) has published 2020 Guidelines based on the best science currently available (7).

For frequencies <6 GHz, the EMFs penetrate deep into tissue (and thus require depth to be considered). Hence, it is useful to describe this in terms of “specific energy absorption rate” (SAR). SAR is the power absorbed per unit mass (W kg^−1^).

For frequencies >6 GHz, the EMFs are absorbed more superficially, so the depth is less relevant. Hence, it is useful to describe exposure in terms of density of absorbed power over area (W m^−2^), which we refer to as “absorbed power density” (Sab).

SAR: The ICNIRP 2020 Guidelines recommend, for EMF exposure from 100 kHz to 300 GHz, a SAR limit of 2 W/kg averaged over 10 g of human tissue (for the head and trunk) for the general public (7). Based on the precautionary principle, several countries adopt more stringent exposure limits than international standards (14). In India, from 2013, mobile handsets permitted to be manufactured or imported are those with an SAR value of 1.6 W/kg. The ICNIRP additionally specifies a whole-body average SAR restriction of 0.08 W/kg for the general public (7).

Sab: The ICNIRP 2020 Guidelines for basic restrictions for EMF exposure from 100 kHz to 300 GHz specify a local Sab of 20 W m^−2^ for the general public (7).

Other measures of RF EMF exposure are whole-body SAR, local SAR, absorbed power density (Sab), specific energy absorption (SA), absorbed energy density (Uab), and electric field (E-field).

Guidelines for limiting RF EMF exposure are holistic. For any given RF EMF frequency, restrictions for all above measures must be met simultaneously (7).

AI for lower SAR/Sab levels: Machine-learning models on mobile handsets effectively optimize antenna tuning to reduce radiation absorption near the human body.

The cable earpiece/speaker mode is a safer and more sensible solution. A useful strategy is to ensure that the mobile telephone operates only when it is at a certain distance from the body. AI should be utilized for this purpose, helping to accustom the user and provide alerts. AI’s real-time analysis and instant decision-making capabilities can provide timely prompts.

Wireless gadgets used in various fields, such as mobile communication, health monitoring, exercise monitoring, athletics, and warfare telecommunications have grown substantially in recent years. There have been significant advancements in wearable antennas for wireless body area networks (WBANs). A major obstacle that must be overcome is the creation of smaller, more powerful antennas (15) that require less radiation. SAR is a parameter that is likely to be monitored for safety concerns, and should be as low as possible for any antenna to ensure the minimum risk to human health (16).

Automatic information delivery between multiple smart applications in wireless networks simultaneously is required, and AI use for this safely is progressing professionally. Reinforcement learning algorithms, such as Q-learning and deep Q networks (DQNs), can make sequential decisions in dynamic environments. AI-enabled wireless networks have recently caught the attention of researchers in academia and industry.

### 2.2 Risk-reducing engineering and environment

An understanding of engineering aspects of human exposure to EMF is necessary for risk reduction. A notable impact on human exposure depends on the channel condition between the base station (transmitting station) (BS) and user equipment (UE).

As the distance between the base station and the user increases, the EMF intensity decreases rapidly. Buildings introduce a shielding effect that attenuates exposure from outdoor base stations.

Strategies to provide a better service level and also reduce the exposure levels over the territory (including sensitive places), as compared to current cellular networks, are: (i) adoption of multiple-input multiple-output (MIMO), (ii) beamforming, (iii) large intelligent surfaces, and (iv) narrow-beam free-space optical (FSO) communication.

Of particular interest is the IEEE 2061–2024 standard for reaching the remotest! It provides affordable broadband access, with low mobility and energy efficiency. It is also aptly referred to as the ‘Frugal 5G network’. Its architectural features (local routing within the access network, macro-BS plus Wi-Fi offload, reduced backhaul) plausibly reduce aggregate transmitted power and therefore incidental exposure (17).

The macro-BS in IEEE-2061 is an advancement that is both safe and advantageous. It is built with any cellular technology capable of supporting a large coverage area. In this, the macro-BS provides large-area coverage but possibly a lower data rate. High-speed connectivity is provided with Wi-Fi deployment. The following *strategies of ‘IEEE-2061’* are useful for minimizing radiation & exposure:

- *Internet*: Access network (AN) for internet connection directly, avoiding the centralized Core Network (CN).
- *Communication*: IEEE-2061 network provides for communication between nearby users with direct routing within AN, and avoiding the CN.

IEEE 2061–2024 is important in the upcoming 6G era, with advantages of a flexible, scalable, and modular network.

#### 2.2.1 Flora fauna

The harm to flora and fauna from new and strong signaling used in 5G/6G technology needs consideration and caution. RF radiation exposure standards adopted by governments and agencies worldwide do not take wildlife into consideration. Many species of flora and fauna, because of distinctive physiologies, have been found to be sensitive to exogenous electromagnetic fields (EMFs) in ways that surpass human reactivity (18). Such exposures may now be affecting endogenous bioelectric states in some species (19). AI should be utilized for guiding and analyzing growing research data. Transmission networks should be suitably placed to cause no or limited environmental damage. Further, wildlife-specific radiation exposure guidelines need to be developed, with robust research programs.

Another important strategy suggested is “Fast transmission from 5G/6G network can be caught centrally and relayed by optic fiber cables.” Underground optic fiber cables will reduce radiation exposure. This strategy should be used whenever connections are needed in fixed sites, such as schools, libraries, workplaces, hospitals, houses, public buildings, public parks, and all new buildings (10).

Satellite direct-to-device (D2D) communications: Furthermore, direct smartphone access to satellites appears fanciful, but is not favorable. First, connecting a low Earth orbit satellite from a handheld device requires the user equipment to transmit at greater power than to a terrestrial base station. This happens as the power of the signal falls off in proportion to the square of the distance traveled, and even low earth orbit (LEO) satellites will be much farther away than ground stations.

Second, gateway earth stations remain necessary in current and planned direct-to-cell architectures. Thus, ground stations’ radiation risks are not eliminated.

Satellite D2D advantages include providing connectivity to remote, rural, or disaster-affected areas. Thus, emergency response in hard-to-cover areas is a boon (20).

Starlink, serving over 2 million active customers in the U.S., is encouraging safe excellence (21, 22). Its advantage is high-speed, low-latency broadband internet. It utilizes a massive network of thousands of LEO satellites to deliver to remote or underserved areas. It requires installing a compact, portable rectangular dish for connecting to the overhead satellites to provide internet access. For mobile telephony, video calls are available.

AI for fully intelligent networks: 6G and beyond wireless communication networks aim is to fulfill the requirements of a fully connected world. This requires the network to be densely deployed. Safety with reduced radiation requires networks to be dynamic, extremely heterogenous with tight quality of service. All this is possible with AI, machine learning (ML), and deep learning (DL). Adaptive network intelligence capable of operating under uncertainty in highly dynamic environments will use AI as computational intelligence (CI) for continual adaptation, autonomous decision-making, and robust control (23, 24).

Radio conditions change, interference, and spectrum availability are dynamic, and autonomous adaptation with AI will be workable. This will lead to more flexible and intelligent use of available radio resources (9).

AI insights in 5G/6G network deployments and functioning will maximize resource usage, minimize energy usage, and promote green communication grandly. A conceptual framework of AI-driven 6G networks has summarized the anticipated advantages as (i) intelligent resource allocation, (ii) dynamic spectrum management, (iii) autonomous network orchestration, and (iv) security and predictive maintenance (25). All of these will further boost safety.

AI is revolutionizing how we approach safety measures across various industries, including healthcare and nuclear energy. It should be utilized for telecommunications, given the widespread use. Its use in real-time exposure modeling should guide infrastructure development. Its potential is to enhance radiation protection by enabling real-time monitoring, simultaneous predictive analysis, and immediate automated response systems (26).

### 2.3 Risk-reducing personalization/habits

**Figure 2:**
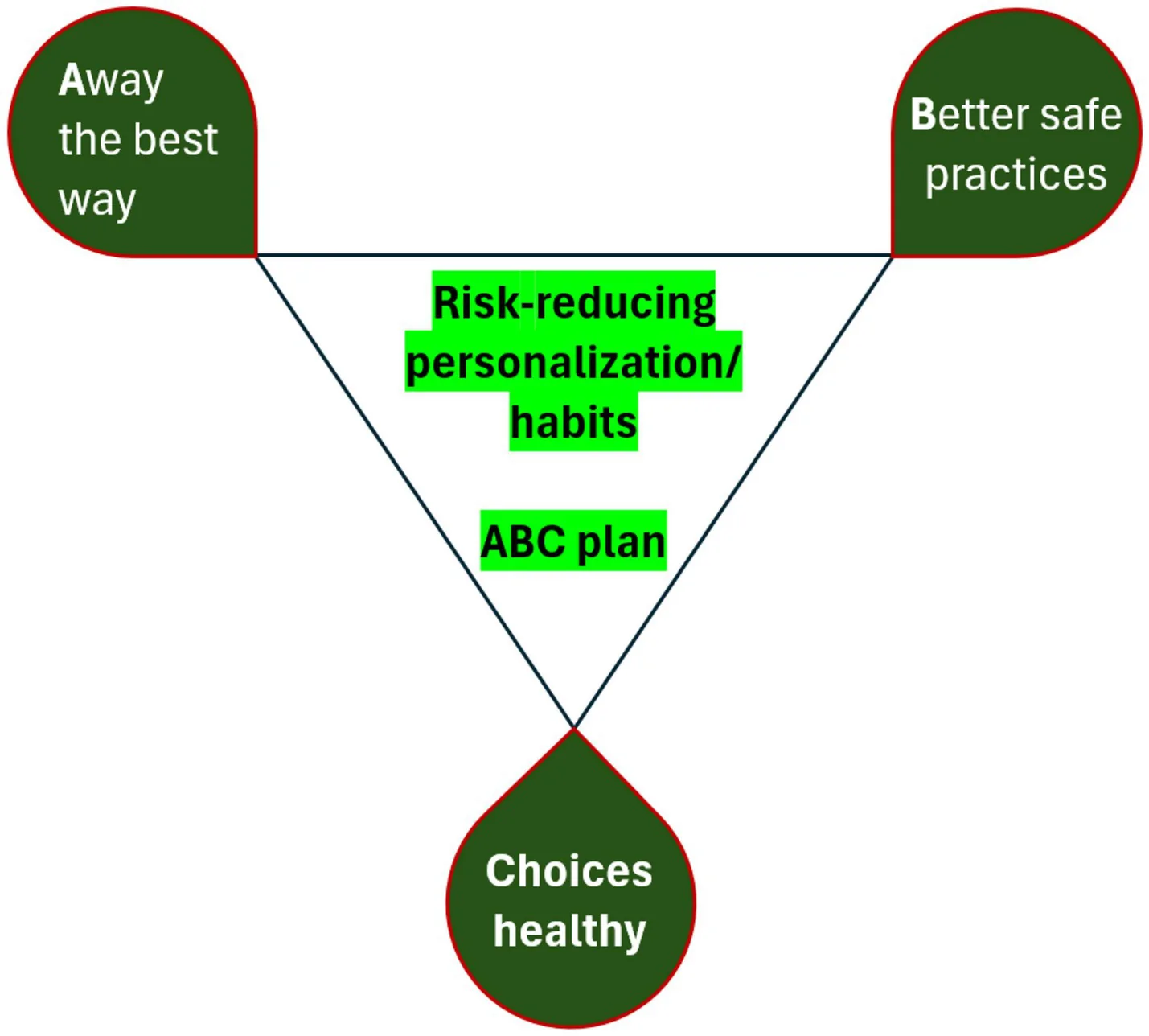
Professional steps fruitful for personalized safety.

Simple steps for lifelong healthy smiles are enumerated as the ‘ABC’ plan (Figure 2). These are consistent and defensible precautionary measures under the As Low As Reasonably Achievable (ALARA) principle.

#### 2.3.1 Away is the best way

Radio frequency (RF) energy decreases in inverse proportion to the square of the distance from the source.

1. Keep distance—Cell phone held away from body to the maximum extent possible.
2. Use a headset (wired/Bluetooth) to keep the handset away from your head.
3. Pressing the phone handset against the head should be totally avoided.
4. Active medical implants: people with these should preferably keep the cell phone at least 15 cm/half foot away from the implant.

#### 2.3.2 Better safe practices

5. Limit the length of mobile calls, for individual/environment safety and sustaining economies.
6. Text should be used as compared to voice, wherever possible.
7. Phone should be used where reception is good. This is because when the radio signal is weak, the mobile phone increases its transmission power. Finding a strong signal and avoiding movement are beneficial.
8. Call connecting: the communication at the start is with higher power, and then the power reduces to an adequate level. Hence, allowing the call to connect and then putting the handset to your ear or starting to speak/listen is beneficial. This will reduce exposure, promoting safety by avoiding more power being radiated during the call connection time.

#### 2.3.3 Choices healthy

9. Mobile Handset SAR: this should be checked at purchase. It should be as per the government’s safety standards and minimum. All this information is available on the internet.
10. Landline (wired) phone should be used when available, avoiding the radiation risks of mobile phones.

AI analysis insights from user behavior patterns and preferences data can guide safe use, and should be put to use personally, with built-in software. Recently, a mobile application (app)-based recommendation system has been developed and tested. In this, smartphone usage patterns are analyzed and generative artificial intelligence (GAI)-based alerts are generated for user recommendations. The results show usefulness in precisely identifying and alerting the users (27).

## 3 Limitations

The presented perspective/conceptual analysis has limitations. These are (i) the conceptual nature of the framework. Although this covers all aspects comprehensively, as we progress, more safety applications with AI should come into picture and practice. As the infrastructure is developed, it should be guided by AI real-time exposure modeling. (ii) There is uncertainty surrounding long-term biological effects. Hence, we have presented safe strategies minimizing exposure, which are desirable. (iii) There is dependence upon future regulatory adoption, but we strongly suggest serious thought should be given to our rationale recommendations.

## 4 Public health readership caution

Our perspective has precautionary behavioral advice, and it is necessary to mention that these themselves can cause adverse psychological and behavioral side effects. These may be anxiety, depression, frustration, feelings of guilt, nocebo effects, etc. Furthermore, attention can be displaced from established risks such as distracted driving, sleep displacement, and problematic smartphone use. Self-jurisdiction is advised in using our advice.

## 5 Future research priorities

(i) EMF exposure dosimetry for high-frequency bands, including dynamic monitoring; (ii) Internet of Bio-Nano-Things (IoBT) particularly for shallow penetration depth effects; (iii) research for promoting safety with dynamic, non-uniform intelligent fields rather than static, uniform background fields; (iv) epidemiological tracking with cohort studies with interdisciplinary collaboration; and (v) AI use should be robust and risks free, including algorithmic bias, unprecedented cybersecurity risks, data privacy breaches, and unvalidated autonomous AI clinical decisions. As AI evolves, regulations should guide and monitor for sound and safe innovations (28).

## 6 Conclusion

We need to prioritize health safety over economics and everything else in the rollout of all radiofrequency technologies. Strategies suggested should be useful for engineering progress, government policies and regulations, and people’s healthy habits.

“*Safety always surely,*

*Artificially Intelligence wonderful working ways protectively,*

*Connected intelligently risk freely.*”

## Data Availability

The original contributions presented in the study are included in the article, further inquiries can be directed to the corresponding author.

## References

[1] Jain S. Radiation in medical practice & health effects of radiation: rationale, risks, and rewards. J Family Med Prim Care. (2021) 10:1520–4. doi: 10.4103/jfmpc.jfmpc_2292_20, 34123885 PMC8144773

[2] WHO Health Topics. Electromagnetic Fields. Available online at: https://www.who.int/india/health-topics/electromagnetic-fields (Accessed March 17, 2026).

[3] International Agency for Research on Cancer (IARC) Working Group on the Evaluation of Carcinogenic Risks to Humans. Non-Ionizing Radiation, Part 2: Radiofrequency Electromagnetic Fields. Lyon (France): IARC; (IARC Monographs on the Evaluation of Carcinogenic Risks to Humans, No. 102). (2013). Available online at: https://www.ncbi.nlm.nih.gov/books/NBK304630/ (Accessed August 17, 2026). PMC4780878 24772662

[4] Karipidis K Baaken D Loney T Blettner M Brzozek C Elwood M . The effect of exposure to radiofrequency fields on cancer risk in the general and working population: a systematic review of human observational studies - part I: most researched outcomes. Environ Int. (2024) 191:108983. doi: 10.1016/j.envint.2024.108983 39241333

[5] IARC Monographs on the Identification of Carcinogenic Hazards to Humans. Report of the Advisory Group to Recommend Priorities for the IARC Monographs during 2025–2029. Available online at: https://monographs.iarc.who.int/wp-content/uploads/2024/11/AGP_Report_2025-2029.pdf (Accessed July 27, 2026).

[6] SCHEER (Scientific Committee on Health, Environmental and Emerging Risks), Final Opinion on the need of a Revision of the Annexes in Council Recommendation 1999/519/ EC and Directive 2013/35/EU, in view of the latest scientific Evidence Available with Regard to Radiofrequency (100kHz–300GHz), Adopted by Written Procedure (2023)

[7] International Commission on Non-Ionizing Radiation Protection (ICNIRP) (2020) ICNIRP Guidelines on Limiting Exposure to Time-Varying Electric, Magnetic and Electromagnetic Fields (100kHz to 300 GHz). Available online at: https://www.icnirp.org/cms/upload/publications/ICNIRPrfgdl2020.pdf (Accessed March 17, 2026)

[8] Chataut R Nankya M Akl R. 6G networks and the AI revolution-exploring technologies, applications, and emerging challenges. Sensors (Basel). (2024) 24:1888. doi: 10.3390/s24061888, 38544151 PMC10975185

[9] Kouhalvandi L Matekovits L. On the role of artificial intelligent technology for millimetre-wave and terahertz applications. Sensors (Basel). (2025) 25:5502. doi: 10.3390/s25175502, 40942934 PMC12431012

[10] Jain S Jain PK. 5G Technology for healthcare and its health effects: wonders, dangers and diligence. J Fam Med Prim Care. (2022) 11:6683–6. doi: 10.4103/jfmpc.jfmpc_1426_22, 36992991 PMC10041185

[11] Simkó M Mattsson MO. 5G wireless communication and health effects-a pragmatic review based on available studies regarding 6 to 100 GHz. Int J Environ Res Public Health. (2019) 16:3406. doi: 10.3390/ijerph16183406, 31540320 PMC6765906

[12] Bordage G. Conceptual frameworks to illuminate and magnify. Med Educ. (2009) 43:312–9. doi: 10.1111/j.1365-2923.2009.03295.x, 19335572

[13] Rougas S Berry A Bierer SB Blanchard RD Cianciolo AT Colbert-Getz JM . Applying conceptual and theoretical frameworks to health professions education research: an introductory workshop. MedEdPORTAL. (2022) 18:11286. doi: 10.15766/mep_2374-8265.11286, 36568035 PMC9715823

[14] Chiaraviglio L Elzanaty A Alouini MS. Health risks associated with 5G exposure: a view from the communications engineering perspective. IEEE Open J Commun Soc. (2021) 2:2131–79. doi: 10.1109/OJCOMS.2021.3106052

[15] Asokan S Kaliappan K. Revolutionizing healthcare with metamaterial-enhanced antennas: a comprehensive review and future directions. Frequenz. (2024) 78:1–9. doi: 10.1515/freq-2023-0236

[16] Rajan VGS Kaliappan K Natarajan SK. SAR reduction techniques for WBAN and mobile applications. Frequenz. (2023) 77:525–36. doi: 10.1515/freq-2022-0297

[17] IEEE Xplore "IEEE Approved Draft Standard for Architecture for Low Mobility Energy Efficient Network for Affordable Broadband Access," in IEEE P2061/D1.1. (2024), 1–97

[18] Levitt BB Lai HC Manville AM. Effects of non-ionizing electromagnetic fields on flora and fauna, part 3. Exposure standards, public policy, laws, and future directions. Rev Environ Health. (2021) 37:531–58. doi: 10.1515/reveh-2021-0083, 34563106

[19] Nyberg NR McCredden JE Weller SG Hardell L. The European Union prioritises economics over health in the rollout of radiofrequency technologies. Rev Environ Health. (2022) 39:47–64. doi: 10.1515/reveh-2022-0106, 36129168

[20] Farrar T. Satellite Direct-to-Device: A Supplement for Terrestrial Cell Coverage White Paper. Available online at: https://wia.org/wp-content/uploads/2025/05/TMF-White-Paper-on-Satellite-D2D_October-2025.pdf (Accessed July 27, 2026).

[21] Yuan A Yang Z Sun Z. Evolution of satellite communication systems toward 5G/6G for 2030 and beyond. Engineering. (2025) 54:1–13. doi: 10.1016/j.eng.2025.06.025

[22] PC MAG Spacex: Starlink is Serving Over 2 Million Active Customers in the U.S. Available online at: https://www.pcmag.com/news/spacex-starlink-has-crossed-2-million-active-customers-in-us (Accessed January 30, 2026)

[23] Arshad R. Muzzammel R. (2023) Realizing intelligence in 6G communications: a review. 2nd international conference on emerging trends in electrical, control, and telecommunication engineering (ETECTE). Lahore. (2023) 1–5.

[24] Lall S Nyathi T Pillay N. Computational intelligence for 6G networks: A survey of machine learning and optimization for enabling technologies and architectures. J Netw Comput Appl. (2026) 253:104533. doi: 10.1016/j.jnca.2026.104533

[25] Balaji CG Menaka S Rajeswari G Ponnusamy S. A unified AI-driven framework for quantum-secured 6G THz networks with intelligent reflecting surfaces and federated edge learning. Sci Rep. (2025) 15:42510. doi: 10.1038/s41598-025-26510-2, 41309772 PMC12660768

[26] Refahi S Shahi M Davaridolatabadi N. A revolution in radiation protection in modern life. J Biomed Phys Eng. (2024) 14:209–10. doi: 10.31661/jbpe.v0i0.2401-1713, 38628896 PMC11016828

[27] Hansda S. Saha R. Misra S. Generative AI-based health hazard prediction from smartphone usage, GLOBECOM 2024–2024 IEEE global communications conference, Cape Town (2024) 3527–3532.

[28] Jain S Jain PK Puranik AK. Digital health technology & cancer care: conceptual framework leading comprehensive fruitfulness. J Healthc Leadersh. (2024) 16:525–35. doi: 10.2147/JHL.S486263, 39679178 PMC11645248

